# Getting there: How commuting time and distance impact students’ health

**DOI:** 10.1371/journal.pone.0314687

**Published:** 2024-12-06

**Authors:** Nattanicha Chairassamee, Kanokwan Chancharoenchai, Wuthiya Saraithong

**Affiliations:** Department of Economics, Kasetsart University, Chatuchak, Bangkok, Thailand; University College Dublin, IRELAND

## Abstract

This study investigates the impact on Thai students’ health of commuting to school, both in terms of time and distance. The individual-level dataset used in this study is obtained from the National Statistical Office (NSO) and limited to students aged from 9 to 18 years old, with 25,461 respondents. While the data were collected in 2016, with mostly unchanged commuting behaviors of Thai students, our results can reflect current health impacts from school commutes. The data indicate that traffic in Bangkok causes students to commute longer to schools than in other provinces. The results from the ordered logistic regression consistently show that commuting time has stronger negative impacts on health than commuting distance does. In other provinces, our results show that long commuting time and distance negatively affect physical and mental health of students. The present study also indicates that investigating either commuting distance or commuting time could bias the results in some sequences.

## 1. Introduction

Commuting to school is a daily routine for millions of students worldwide. Depending on the mode of transportation, distance, and time, the school commute can have a significant impact on students’ physical and mental health. This raises the question of how much time spent on the road is appropriate for students.

In recent years, there has been growing concern about the negative impacts of sedentary behavior on health outcomes. A long school commute can contribute to increased sedentary behavior, as students may spend a significant amount of time sitting in a car or on public transportation. This can negatively impact physical health outcomes such as obesity, and blood pressure [1].

Furthermore, commuting can also have negative impacts on mental health outcomes. For example, students may experience stress, anxiety, or depression related to commuting, particularly if it involves a long distance or unreliable transportation options [2, 3]. Additionally, students with longer commutes may have less time for other activities that promote mental health, such as spending time with friends and family, participating in extracurricular activities, or engaging in leisure activities.

While there is growing recognition of the importance of promoting active and healthy commuting among students, for example, [4, 5], further research is needed to better understand the specific effects of commuting on students’ health. Given the ubiquity of commuting for students, understanding its health implications is crucial for developing effective interventions to promote active and healthy commuting behaviors and improve overall health outcomes.

**Fig 1:**
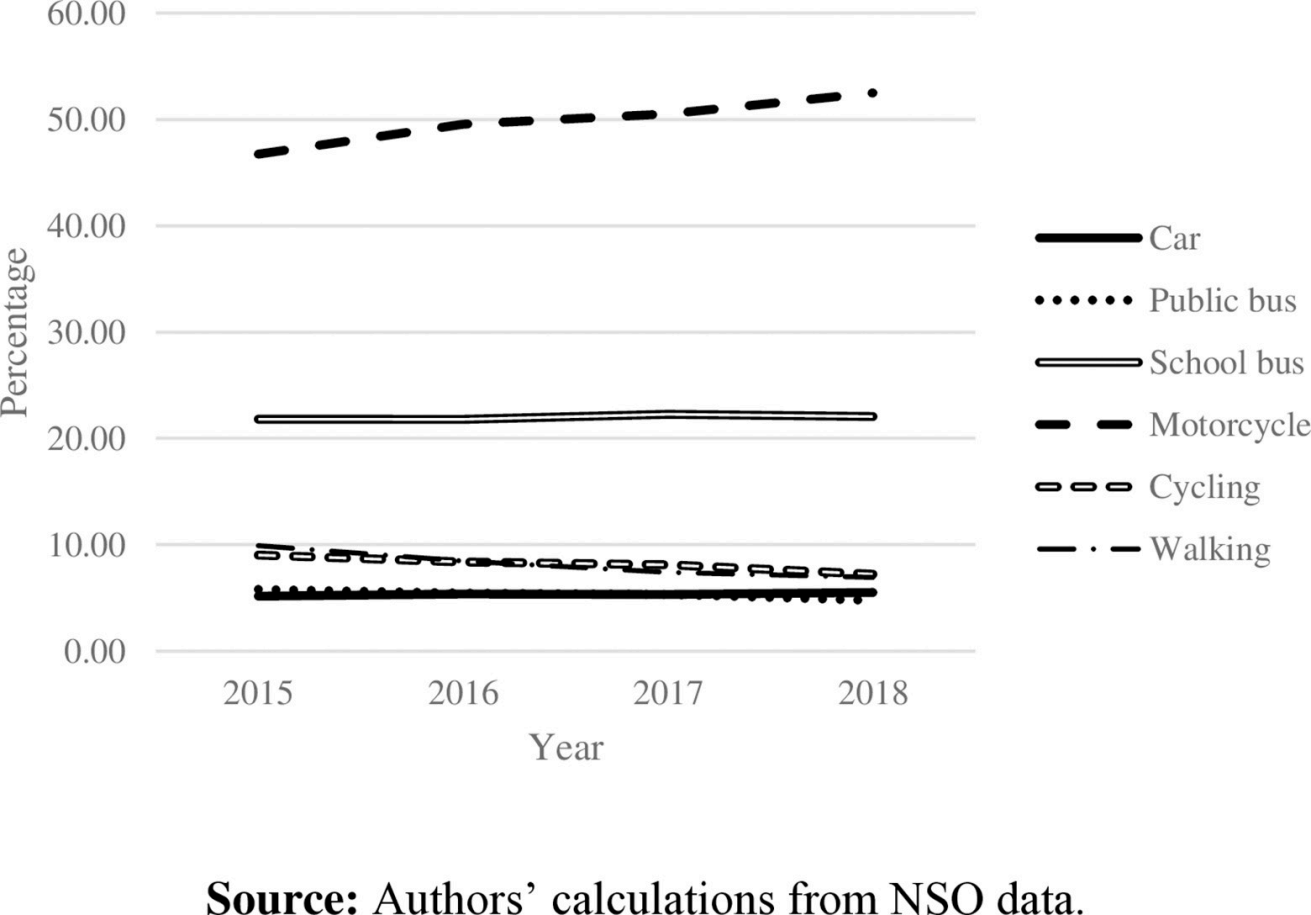
Vehicle percentage used by Thai students. **Source:** Authors’ calculations from NSO data.

**Fig 2:**
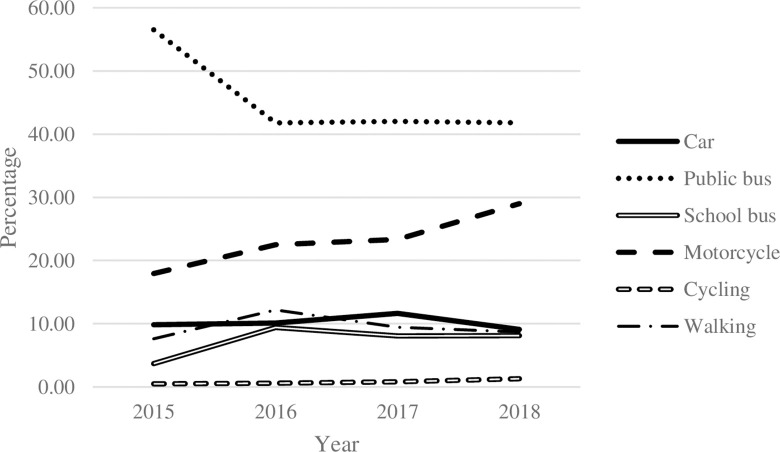
Vehicles percentage used by Thai students in Bangkok. **Source:** Authors’ calculations from NSO data.

Commuting can be divided into two groups: passive (i.e., by car, motorcycle, or bus) and active (i.e., walking and cycling) modes. According to the National Statistical Office of Thailand, from 2015 to 2018, passive commuting modes were generally the most frequently used for students. At the same time, only a small proportion of students chose active mode (Fig 1). In Bangkok, approximately 50% of students commute to schools by public bus; however, the data show a downward trend (Fig 2). Commuting by motorcycle became another choice for students in Bangkok. Even though private cars, public buses, and motorcycles are considered on-road commuting modes, specific characteristics of motorcycles cause commuters to spend less commuting time [6]. Consistent with commuting time in Bangkok (Fig 4), the higher the proportion of students taking motorcycles to school, the less time spent on their school commutes. Even though Bangkok has had Skytrain and Subway since 1999, the proportion of students using those modes was less than 5% during the study period and has tended to remain constant over the years.

**Fig 3:**
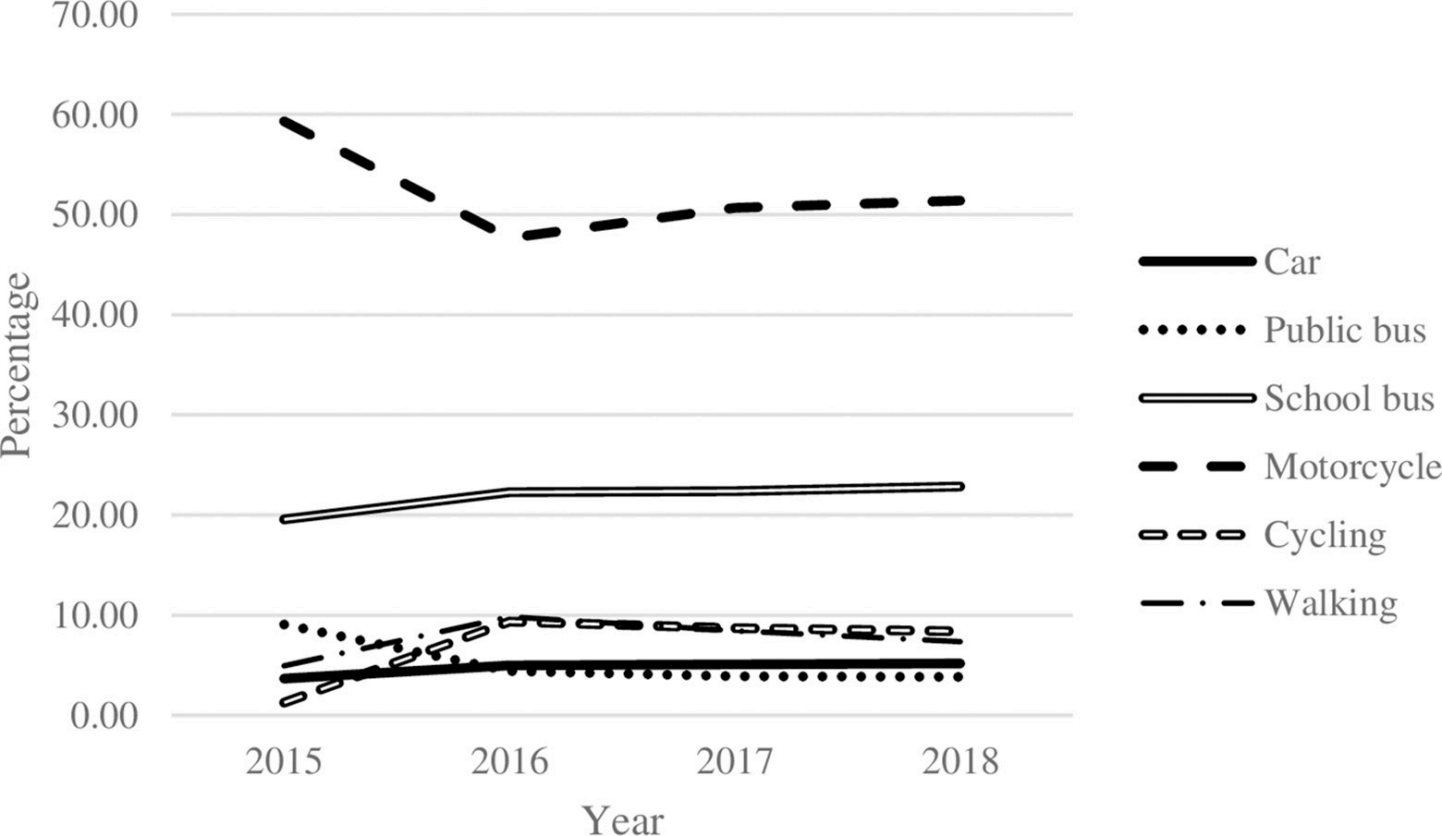
Vehicles percentage used by Thai students in other provinces. **Source:** Authors’ calculations from NSO data.

**Fig 4:**
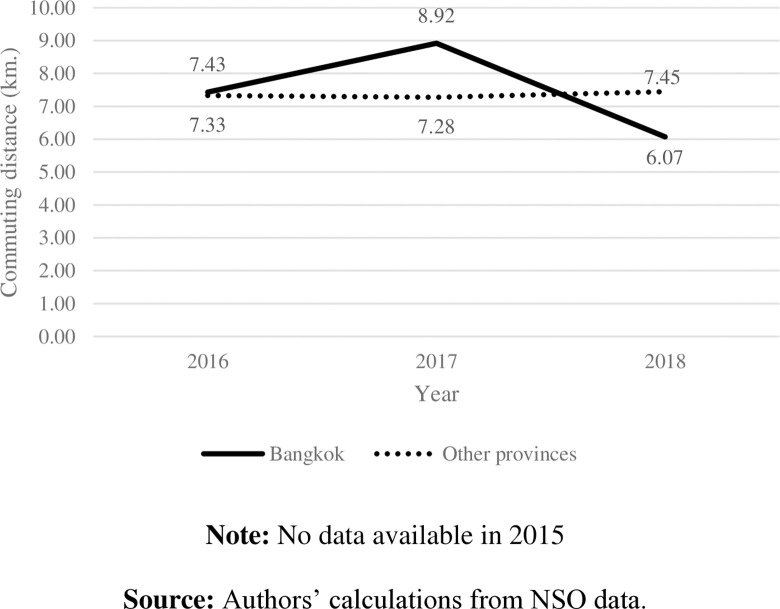
School commuting distance by area. **Note:** No data available in 2015. **Source:** Authors’ calculations from NSO data.

**Fig 5:**
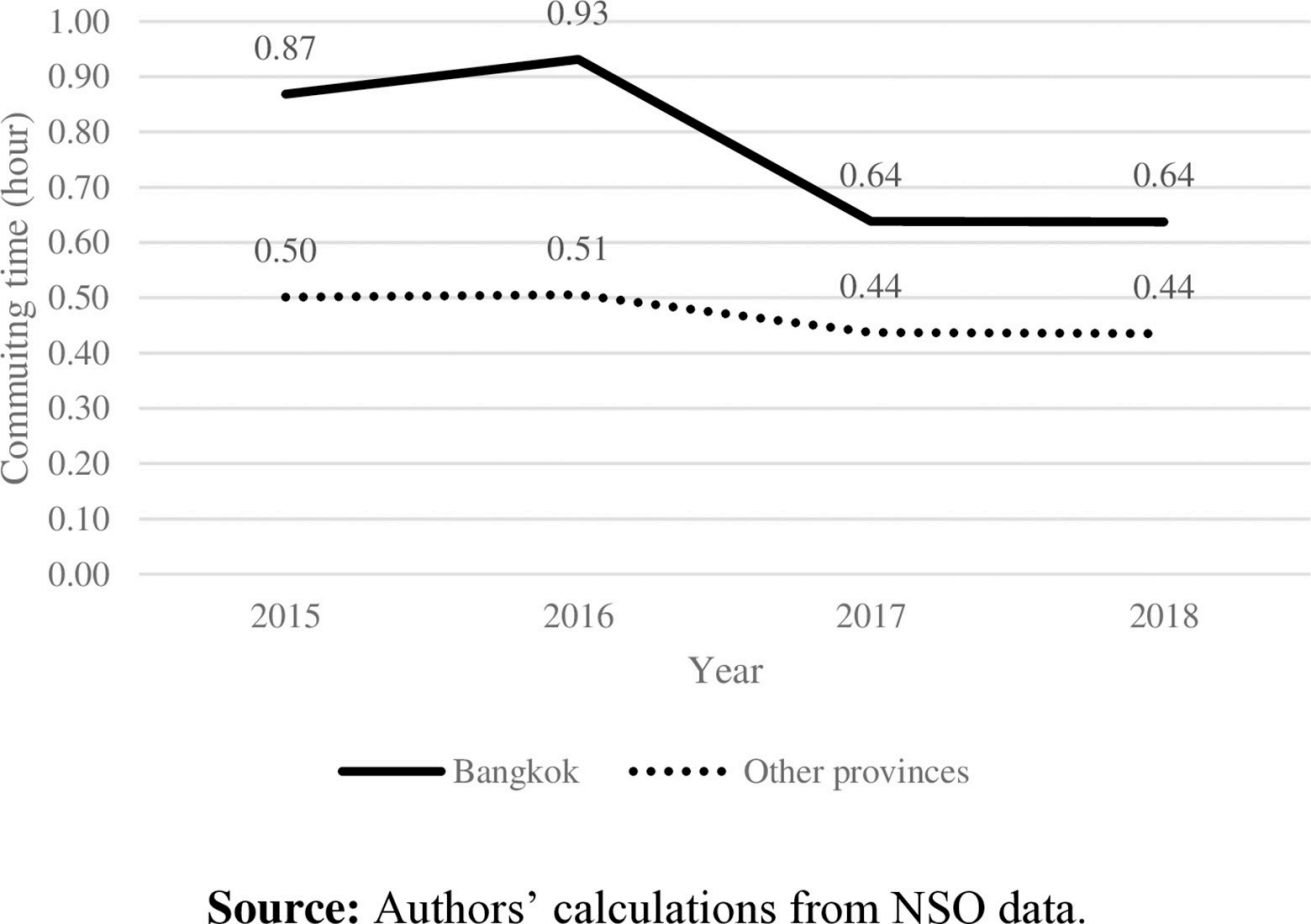
School commuting time by area. **Source:** Authors’ calculations from NSO data.

**Fig 6:**
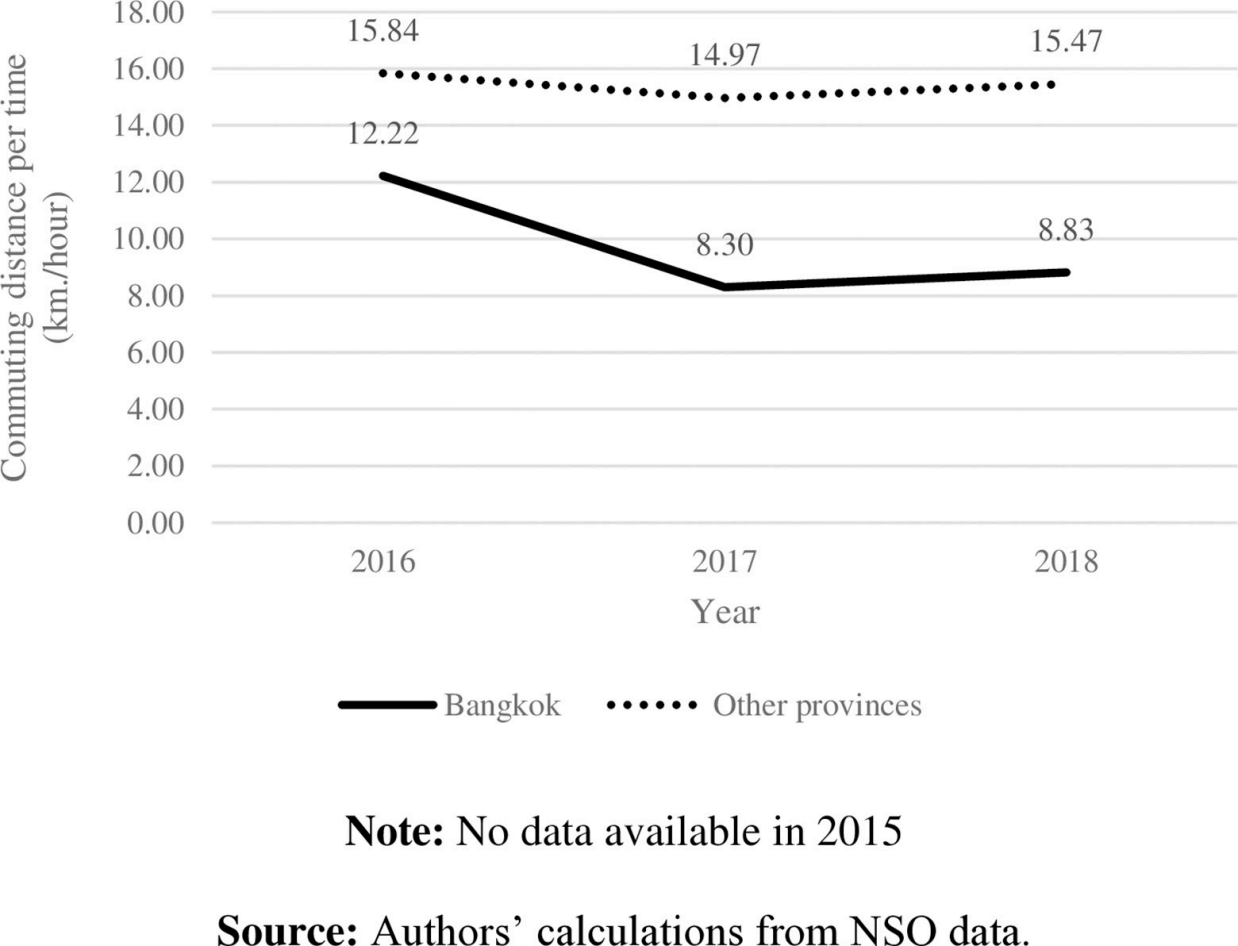
School commuting distance per time by area. **Note:** No data available in 2015. **Source:** Authors’ calculations from NSO data.

Students in other provinces primarily used motorcycles (Fig 3). With a shorter distance to school and less traffic in those areas (Fig 4), students were likely to spend less time on their school commutes (Fig 5) and had higher distance per hour than those in Bangkok (Fig 6).

Even though the data were collected in a short period, they indicated the trend of how Thai students commuted to school, which can reflect their current behaviors. Additionally, these data affirm our hypothesis that there is a low correlation between commuting distance and commuting time in Thailand, particularly Bangkok. To account for traffic and transportation infrastructure issues across areas, this study simultaneously investigates how commuting time and distance to school impact students’ health.

Our paper offers two specific points to examine the evidence for the relationship between commuting time and distance and physical and mental health outcomes, particularly for Thai students.

First, our results show how commuting distance and time jointly affect students’ physical and mental health in Thailand.

Previous studies (e.g., [7–11]) investigate the impacts of commuting time and distance separately. As distinct from prior studies, the present study jointly examines commuting time and distance to account for different traffic conditions and commuting-related structures, such as public transportation and road quality, across areas. Because of those factors, more time spent commuting does not necessarily mean commuting longer distances, as mentioned earlier in Figs 1 to 6. The results from including both commuting distance and time in our models show significant negative effects on health, regardless of the area considered. They also indicate a low possibility of the high/perfect multicollinearity issue. Therefore, omitting either of these variables may bias the results in some sequences.

Second, we examine the impacts of students’ commutes to school, based on where their household is located in relation to school.

A few studies investigate the effects of school commutes by location—i.e., within urban or rural areas (e.g., [2]). As mentioned earlier, traffic and transportation-related infrastructure can vary across areas, affecting students’ commuting behaviors. We exploit the large dataset by deeply investigating commuting effects by home-school location. This allows us to explore school commutes within urban or rural areas and across areas where travel difficulties arise for different reasons. The results show that even students studying in the same area where they live can experience physical and mental health issues, which are not different from those commuting inbound across districts—studying and living in different districts. Commuting affects students in core urban areas, such as Bangkok and other municipalities, similarly to the way it does in rural areas.

The rest of the paper is organized as follows: Section 2 discusses the related literature. Section 3 highlights several empirical facts from the dataset and details the methodology used in this study. Section 4 shows the results of the ordered logistic analysis. Sections 5 and 6 provide study limitations, conclusion, and discussion. Section 7 presents recommendations based on the study.

## 2. Related literature and conceptual framework

### 2.1 Literature review

In general, because of their high correlations, commuting time and distance are separately investigated in most previous studies. The longer the distance, the more time it takes to commute, and vice versa. However, the different commuting time and distance proxies used may raise a concern. Self-estimated commuting time and distance are more likely to be overestimated, particularly for long commutes. The actual commuting time depends on route planners and the average speed of a trip, causing a low correlation between these variables [10].

Longer commuting distances have been found to be associated with a range of negative health issues and other outcomes. [12], for example, have pointed out that short-distance commuters are more likely to be happy commuters, leading them to be more productive. However, only a few studies find no evidence that long commuting distance in general is associated with a lower well-being (e.g., [8]).

Commuting time is more commonly used than commuting distance to investigate the effects of commuting. However, the results from using either commuting distance or time effects are similar. According to [9], individuals who commute to work for more than one hour per day appear to have a statistically higher probability of reporting bad health status than do those who commute for less than one hour.

In terms of mental health and emotions, [11] have found that additional commuting time is positively associated with depression. Prolonged commutes have significant and negative direct effects on overall life satisfaction and happiness [13] and in particular, on family life and leisure time [8]. They can also indirectly affect emotional well-being by impairing health and reducing job satisfaction and community-based social capital [14].

Furthermore, commuting for longer durations can also affect commuters’ overall performance. For instance, in adults, a longer commuting duration lowers productivity by increasing absence due to sickness [15].

Commuting’s effects on student health are similar to those for adults. For undergraduate students, [16] have found that commuting for long duration negatively affects commuting satisfaction. Moreover, commuting durations have an impact on students’ study performance. [17] has indicated that students living farther from their universities are less likely to attend classes than those living closer to campus do.

Most studies in developing countries emphasizing commuting-to-school effects for young children are conducted in China. [2] have shown that longer commutes are negatively associated with children’s psychological well-being and academic achievement. Children living in rural areas took the longest on average one-way trip, while travel time had a greater influence for children living in urban centers and rural areas.

Similarly, in Brazil, [7] have found that long commutes negatively affect teenagers’ wellbeing, regardless of transport mode. Long commutes lead to lower self-reported health and mental health scores, lower grades, less time spent surfing the Internet, playing games, and sleeping, higher BMI and absenteeism, and more time needed to complete household chores. A study by [18] has similarly shown that the duration of commuting can have a negative causal effect on academic achievement.

The negative impacts of long school commutes on children’s health are also found in developed countries. In the United States, [19] have found that school commute time is strongly inversely related to the time spent sleeping, and for those with long commutes, is negatively related to the time spent exercising. Thus, increasing journey-to-school distances may have troubling public health implications for teens. Similarly, [20] surveyed children aged 10 to 15 years. In line with others, they conclude that a shorter journey was experienced as having a higher quality and resulted in more positive feelings. Their findings, however, also show that children traveling for longer durations performed better in the word-fluency test if using their smartphones or doing a combination of activities during their journeys.

Only a few studies find an insignificant relationship between trip duration and mental health, in particular as regards students’ mood and life satisfaction (e.g., [21]).

### 2.2 Literature summary and conceptual framework

**Fig 7:**
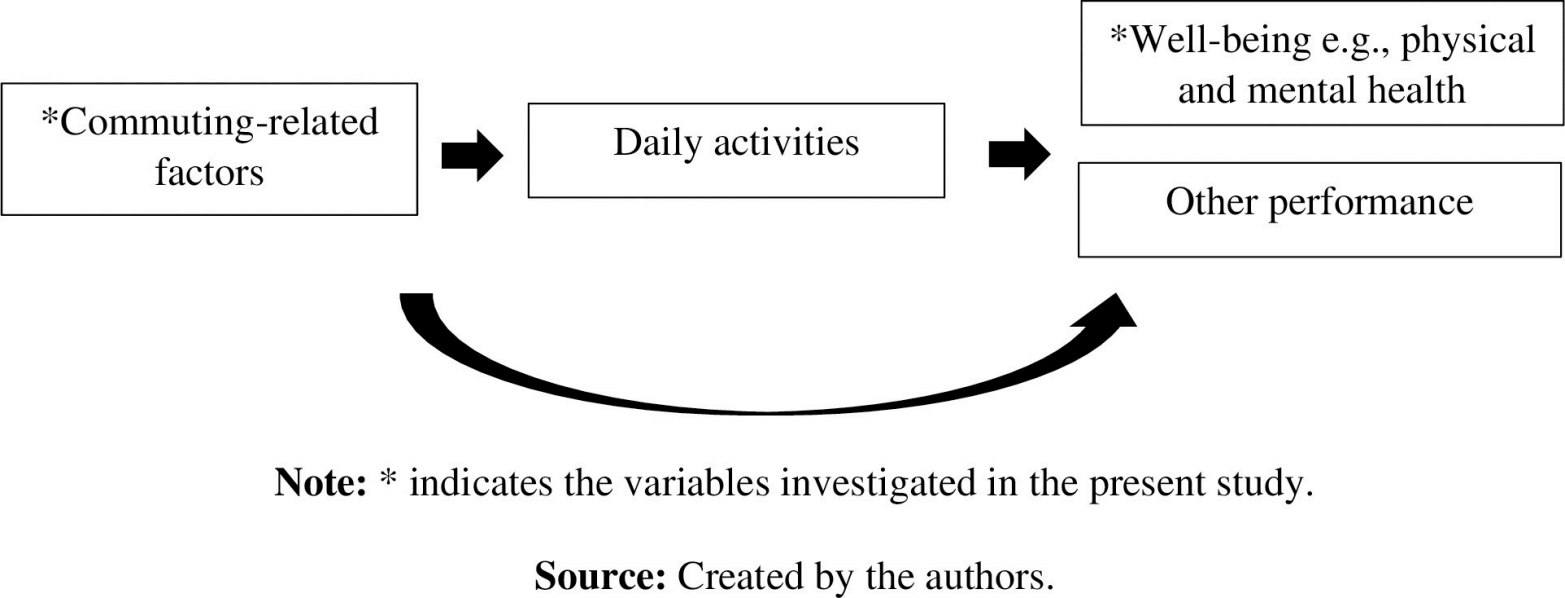
Conceptual framework of commuting effects on well-being. **Note:** * indicates the variable investigated in the present study. **Source:** Created by the authors.

According to the literature review, we can summarize the ways in which commuting affects well-being, as shown in Fig 7. The impact of commuting-related factors on well-being and other outcomes can be indirectly investigated through daily activities, such as sleeping duration and leisure time spent. They can also be directly explored, which is the main purpose of the present study. We mainly investigate how school commutes, in particular commuting distance and time, directly affect students’ health. To shed light more on commuting patterns across areas, we further study the effects of commuting to school according to school-household locations.

## 3. Data and methodology

### 3.1 Data

The present study uses individual data obtained from the National Statistical Organization (NSO) under the migration dataset. Our sample is limited to students aged from 9–18 years old—studying at upper elementary to upper secondary school—to avoid biases from self-evaluation by young children. They were asked to self-assess the impacts of commuting to school on physical and mental health. According to Thai laws and regulations students in this age range are not allowed to have a driver’s license, as such they will be considered passengers for all passive commuting modes.

Unfortunately, the data related to physical and mental health from commutes were collected only in 2016; therefore, this study uses cross-sectional data on 25,461 respondents.

The NSO conducted a self-assessment questionnaire about physical and mental health issues related to commuting. The respondents were directly asked about their health impacts from school commutes, which were rated on a scale from zero to three. Zero indicates no effect on physical or mental health from commuting, while the levels from one to three indicate low, medium, and high negative impacts, respectively.

The dataset used in this study is the secondary data. Since samples are fully anonymized, our study is eligible for a waiver of ethical review.

### 3.2 Descriptive statistics

The descriptive statistics shown in Table 1 indicate that the average rates of physical and mental suffering from school commutes equal 0.11 and 0.14, respectively. Even though both scales are rated lower than one, which implies a low negative health impact, this issue still needs further investigation, to improve students’ well-being. Generally, the students spend around 0.5 hours and commute 6.41 kilometers for a one-way school commute. Most students—16,333 observations, or 66.67%—study at a school located in a municipal area. Only a small proportion—972 observations, or 3.61%—of the samples study at a school located in Bangkok, the capital of Thailand. According to congestion by time, commuting rush hour lasts from 7 AM to 9 AM and 5 PM to 7 PM [22]. More than 90% of those samples commute during rush hour, in the morning, in the evening, or in both. Around 20% of the students sampled commute by an active mode, such as walking or cycling.

**Table 1:** Descriptive statistics.

	Description	Mean	S.D.	Max.	Min.
**Health assessment**					
Physical health	4-scale rating of negative impact on physical health from commuting; no effect, low, medium, and high effect	0.113	0.379	0	3
Mental health	4-scale rating of negative impact on mental health from commuting; no effect, low, medium, and high effect	0.136	0.410	0	3
**Commuting**					
Total commuting time	One-way commuting time (unit: hours)	0.477	0.381	2	0.167
Total commuting distance	One-way commuting distance (unit: kilometers)	6.410	7.790	45	0.001
**Control variables**					
**Sociodemographic**					
Female	1: if the respondents are female/0: otherwise	0.505	0.500	1	0
Age	Respondent’s age in years (unit: years)	13.086	2.777	18	9
Number of household members	Number of family members (unit: persons)	4.476	1.801	25	1
Family income	Family income per month from all sources (unit: thousand Thai baht)	8.289	14.805	249.000	0.000
**Other commuting-related**					
Schools located in Bangkok	1: if the respondents study at a school located in Bangkok/0: otherwise	0.037	0.190	1	0
Schools located in municipalities^#^	1: if the respondents study at a school located in municipalities/0: otherwise	0.667	0.471	1	0
Commuting at rush hour	1: if the respondents commute at rush hour, either in the morning or evening, or both/0: otherwise	0.909	0.288	1	0
Active commuting mode (i.e., walking and biking)	1: if the respondents commute by active mode/0: otherwise	0.195	0.396	1	0

**Notes:** S.D., Max., and Min., respectively, stand for standard deviation, maximum value, and minimum value. ^#^ All areas in Bangkok are classified as municipal areas; therefore, schools located in municipalities exclude schools located in Bangkok. The percentage of students studying at a school located in a municipal area is calculated based on 24,500 respondents.

Approximately 50% of the samples are female and average 13 years old. There are approximately four members in a family, with an average household income of less than THB 8,500 per month.

Before showing the regression results, we first highlight a few empirical facts about overall commuting-to-school patterns and students’ health.

#### 3.2.1 Stylized fact 1: Students live and study in core areas

Since Bangkok has different public transportation modes from other provinces and is an autonomous administrative region, we group together students who study, live, or both in Bangkok, and refer this group as “Bangkok.” The rest of the samples are called “Other provinces.”

**Fig 8:**
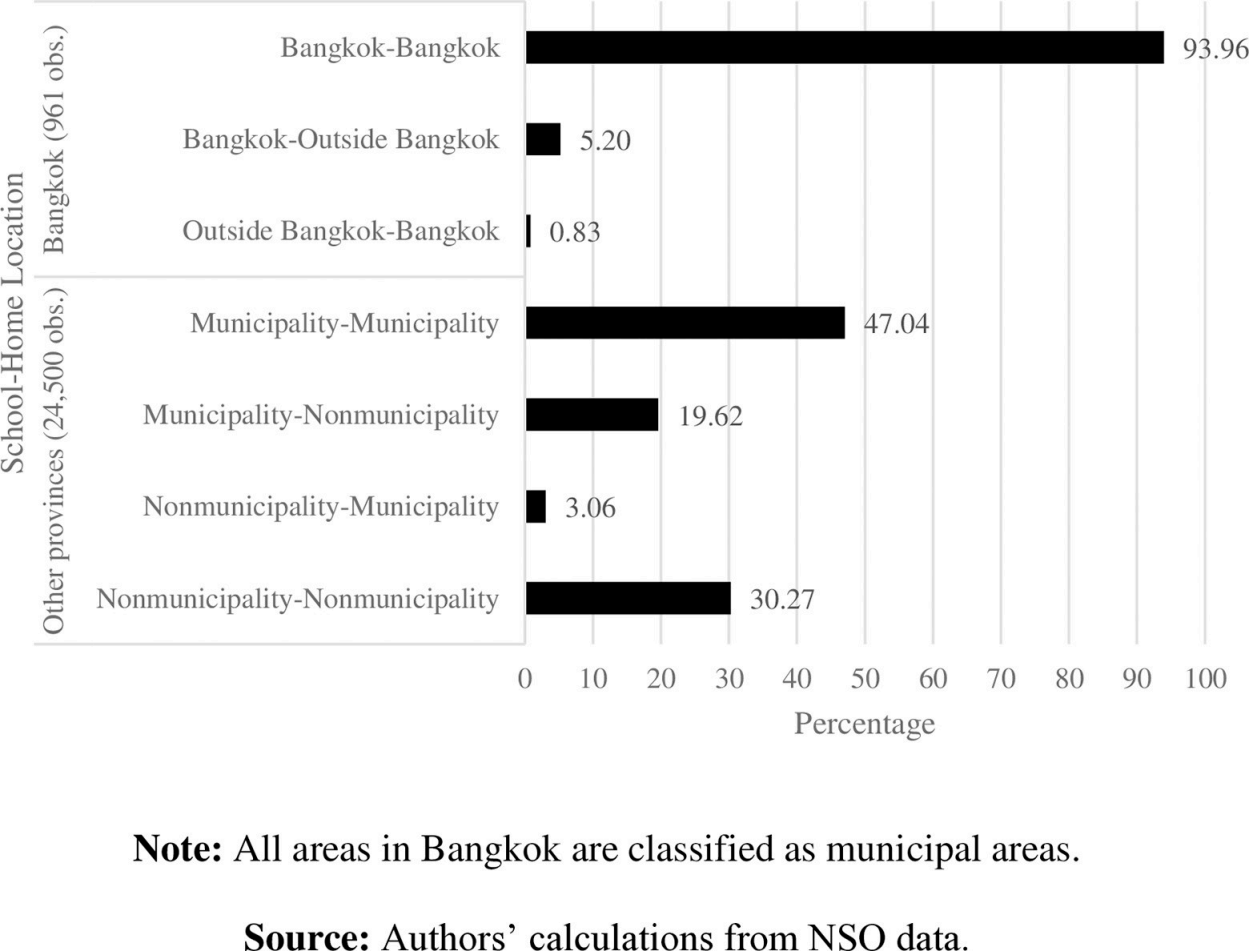
Percentage of students by school-home location. **Note:** All areas in Bangkok are classified as municipal areas. **Source:** Authors’ calculations from NSO data.

In Fig 8, 93.96% out of 961 students study and live in Bangkok. Similarly, students in other provinces tend to study in the same area where they live. Approximately 47% of 24,500 students study and live in municipalities, while 30.27% study and live outside municipalities. These data indicate that most students go to a school located within their home district.

Only a small proportion of students, 3.06%, commute to study outside municipalities. In contrast, 5.20% of students in Bangkok and almost 20% of students in other provinces have to commute to Bangkok or urban areas to study. This is because of the high concentration and variety of schools in central core areas, driving students to commute inbound to Bangkok and municipal areas. It also implies an attitude of parents toward well-known and reputable schools in urban areas (e.g., [23, 24]), leading students to commute across districts from their home to school.

**Fig 9:**
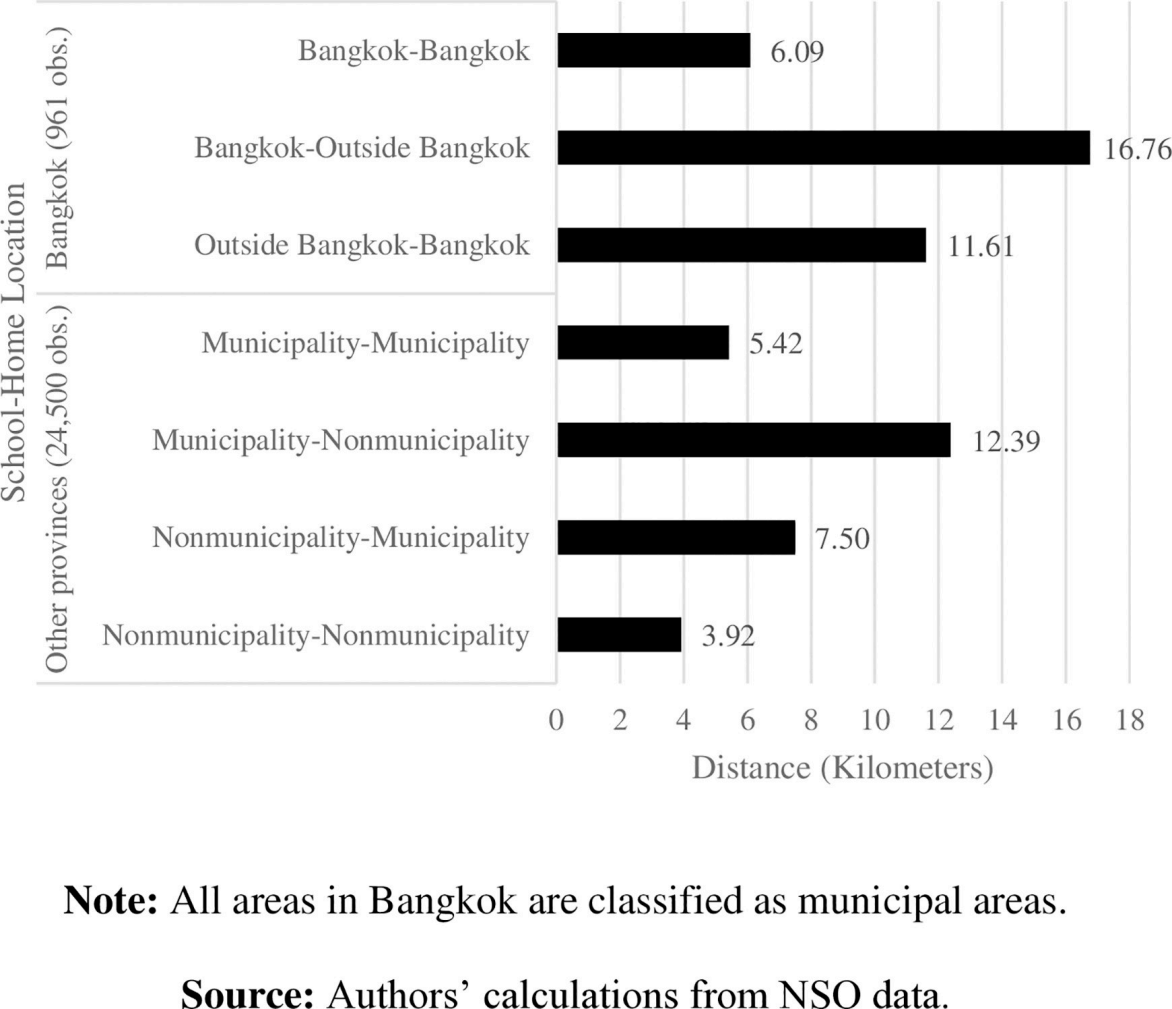
Average commuting distance by school-home location. **Note:** All areas in Bangkok are classified as municipal areas. **Source:** Authors’ calculations from NSO data.

**Fig 10:**
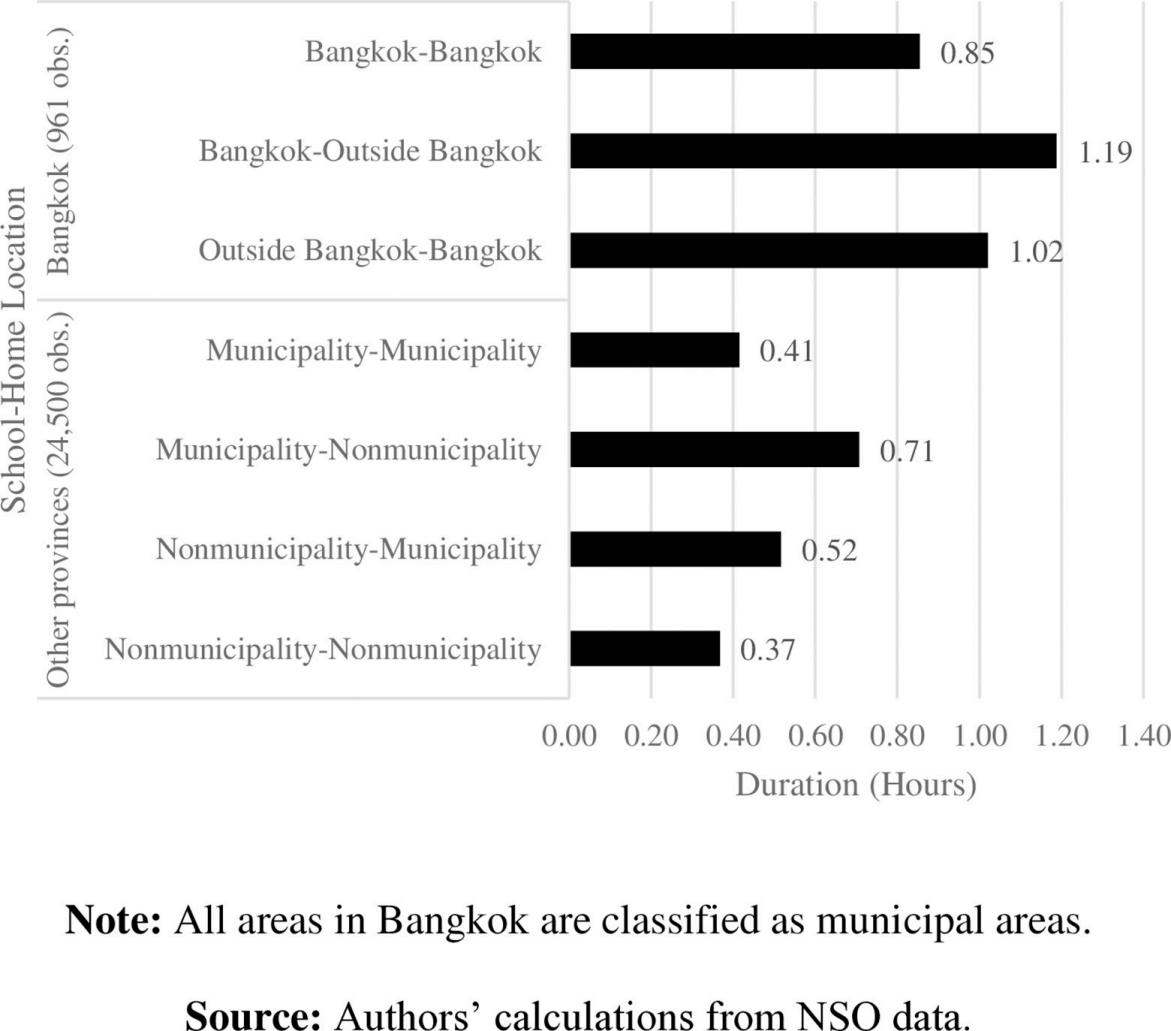
Average commuting time by school-home location. **Note:** All areas in Bangkok are classified as municipal areas. **Source:** Authors’ calculations from NSO data.

**3.2.2 Stylized fact 2: Students commute longer when school and home are in different districts.** Figs 9 and 10 show one-way commuting distance and time according to school-home locations. Generally, students who study and live in different districts of Bangkok commute longer distances, for more time, than those who study and live in same area do. Students living outside Bangkok commute the longest distance—almost three times longer than students who live in Bangkok do—and spend more than an hour for a one-way school commute.

Students who study in municipal areas and live outside their school location obviously commute the longest distance to school, compared to students in other provinces. Students who study in a location where they also live (municipal-municipal or outside municipal-outside municipal) are likely to commute shorter distances and spend less time traveling between their home and school. Unsurprisingly, students studying and living outside municipal areas commute the shortest distances and spend the least time commuting. This fact implies less traffic congestion in rural areas than in Bangkok and municipalities.

**Fig 11:**
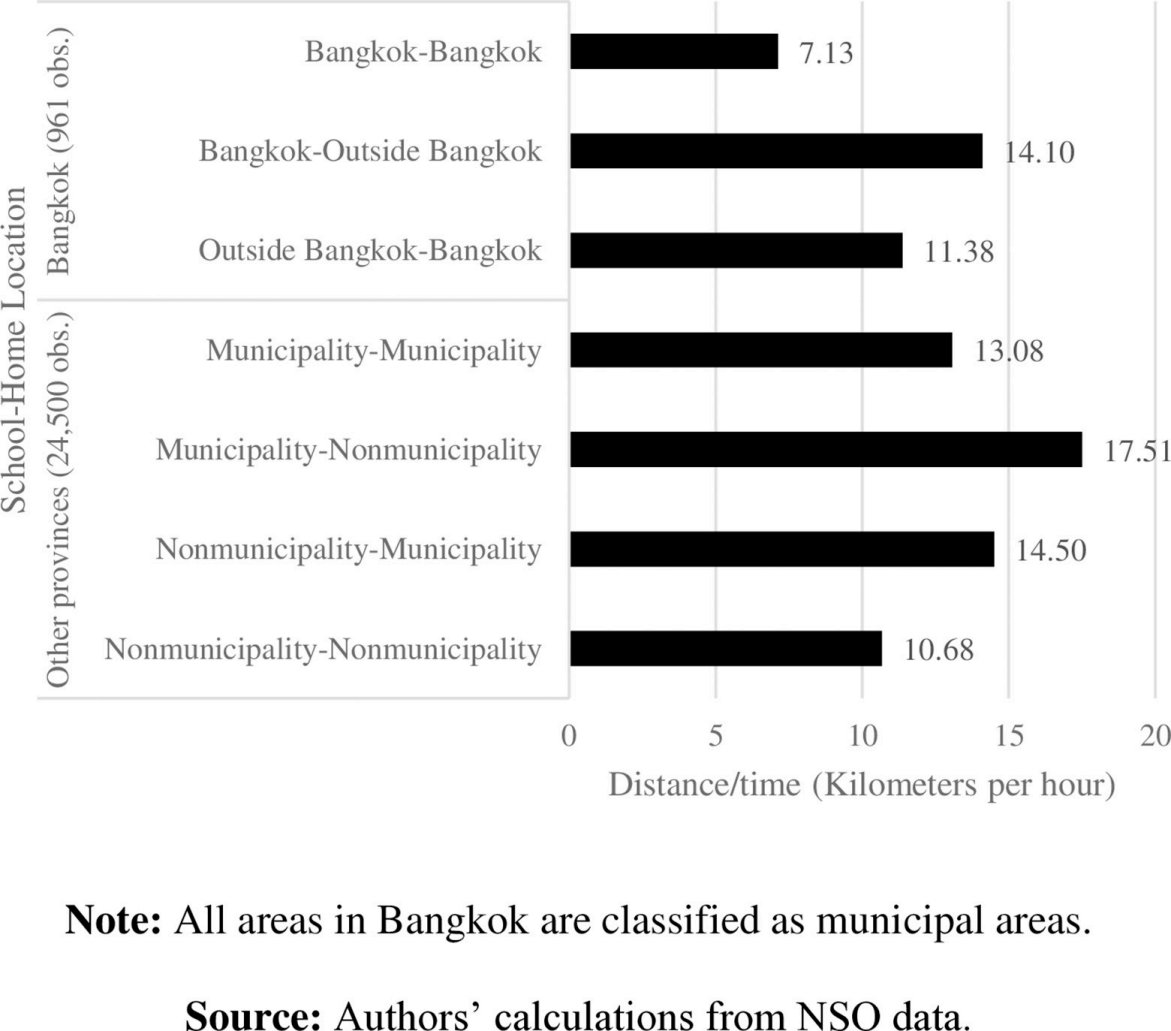
Average commuting distance per hour by school-home location. **Note:** All areas in Bangkok are classified as municipal areas. **Source:** Authors’ calculations from NSO data.

Our data show somewhat contradictory findings to those of [2]. Regardless of whether considering urban or rural areas, Thai students studying and living in the same district are likely to commute shorter distances to school than students who live and study in different districts do. On average, students in other provinces spend less time commuting to school and have higher distance per hour than those students in Bangkok (Fig 11). Altogether, this implies severe traffic in the capital areas. Surprisingly, students who study and live in rural areas of other provinces commute approximately 11 kilometers per hour—the shortest distances compared to other students outside Bangkok. With light traffic in those rural areas, the long time spent commuting to school may result from other factors, such as public transportation and road quality.

### 3.3 Methodology

To address how student health suffers from commuting, an ordered logistic regression is used, since the health assessment is a scale from zero to three, as mentioned in the data section. We include both commuting time and distance to account for different traffic situations and commuting-related structures across areas, as mentioned earlier in the empirical facts. The specification is as follows:

$${Health}_{ip}=\alpha +\beta ({Dist}_{ip})+\gamma ({Time}_{ip})+\delta (X_{ip}^{\prime })+\rho (Z_{ip}^{\prime })+\sigma_{p}+\epsilon_{ip},$$

where *Health*_*ip*_ is a level of either the physical or mental health impact from commuting to school of student *i* in province *p*. Both mental and physical health statuses have four ratings: no effect, and low, medium, and high negative effects. *Dist*_*ip*_ is a one-way commuting distance between home and school, while *Time*_*ip*_ is a one-way commuting duration between home and school. $X_{ip}^{\prime }$ is a set of control variables related to the socioeconomics of student *i*. $Z_{ip}^{\prime }$ is a set of commuting-related control variables. All control variables are reported in Table 1. Even though most students study and live in the same province, some students commute from different provinces, especially municipal provinces within Bangkok metropolitan region. We also add household provincial fixed effect (*σ*_*p*_) to control for unobservables across provinces.

As mentioned earlier in the data section, the impacts on physical and mental health in this study are specifically derived from the school-home commute. Consequently, in our study, there is less possibility of an endogeneity bias between the commuting time and distance, and commuters’ health. In addition, the full control variables reported in Table 1 should alleviate concern about a selection bias resulting from commuting distance. Low family income, for instance, may be located at a place far away from school, forced to commute longer than those with high family income (e.g., [17, 25]).

In S1 Appendix, we also show brief correlations between commuting time and distance. The results indicate, on average, 60% of variables’ correlation. Considering commuting in Bangkok, these two variables have lower correlation than in other provinces, indicating severe traffic in the capital areas. Namely, students may commute short distances with long commuting times.

## 4. Empirical results

The results in this section report only the coefficients and marginal effects of commuting distance and time as our main independent variables. The full results are reported in S2 Appendix. As mentioned earlier, students in “Bangkok” are referred to as students who study or live, or both, in Bangkok. The rest of the samples are called “Other provinces.”

In Bangkok (Panel A of Table 2), commuting distance is significantly negative, meaning that increases in commuting distance reduce mental health issues. In contrast, commuting duration has a strong impact on students’ mental health. Commuting for a longer time increases the likelihood of low to high negative mental health issues. An additional hour on the road can generate 11% more in low and medium mental effects, while increasing high mental effects by 0.7%.

**Table 2:** Commuting effects on mental health.

	Coefficient	Marginal Effects			
		*Y*_*i*_ = 0	*Y*_*i*_ = 1	*Y*_*i*_ = 2	*Y*_*i*_ = 3
		No effect	Low	Medium	High
**A: Bangkok**					
Distance	-0.0262*	0.0055*	-0.0026*	-0.0027*	-0.0002
	(0.0152)	(0.0032)	(0.0016)	(0.0016)	(0.0001)
Time	1.1206***	-0.2349***	0.1121***	0.1150***	0.0077***
	(0.1649)	(0.0328)	(0.0175)	(0.0178)	(0.0029)
Thresholds					
*Y*_*i*_ = 1 (Threshold1)	2.2602	Observations	961		
*Y*_*i*_ = 2 (Threshold2)	3.9964	R^2^	0.0975		
*Y*_*i*_ = 3 (Threshold3)	7.4277				
**B: Other provinces**					
Distance	0.0193***	-0.0015***	0.0012***	0.0003***	0.0000***
	(0.0039)	(0.0003)	(0.0002)	(0.0001)	(0.0000)
Time	0.6531***	-0.0513***	0.0405***	0.0098***	0.0010***
	(0.0797)	(0.0063)	(0.005)	(0.0013)	(0.0002)
Thresholds					
*Y*_*i*_ = 1 (Threshold1)	1.9991	Observations	24,500		
*Y*_*i*_ = 2 (Threshold2)	3.9882	R^2^	0.1412		
*Y*_*i*_ = 3 (Threshold3)	6.4551				

**Notes:** In the ordered logistic regression, the well-being rating ranges from zero to three, in which zero indicates no negative effect and three indicates a high negative effect on health. Robust standard errors are in parentheses. The full results of the ordered logistic regression are reported in S2 Appendix.

*p < 0.10

**p < 0.05

***p < 0.01.

Estimated mental health equal to zero indicates no effect. The thresholds show that estimated mental health ranges between more than zero and less than or equal to Threshold1 ($0<\hat{Y_{i}}\leq Threshold1$) indicate a low effect on mental health (*Y*_*i*_ = 1). Estimated mental health ranges between more than Threshold1 and less than or equal to Threshold2 ($Threshold1<\hat{Y_{i}}\leq Threshold2$) indicate a medium effect on mental health (*Y*_*i*_ = 2). Estimated mental health ranges between more than Threshold2 and less than or equal to Threshold3 ($Threshold2<\hat{Y_{i}}\leq Threshold3$) indicate a high effect on mental health (*Y*_*i*_ = 3).

School-household location may explain the unexpected result of commuting distance on mental health issues. All areas in Bangkok are counted as municipal areas, however; inner/ core/business areas of Bangkok definitely experience worse traffic than outer areas do. Since we do not have data for specific school and household locations in Bangkok, this could generate unexpected results. Students who study in outer areas may commute long distances, but with less traffic congestion than inner areas, those students could experience lower mental effects from their school commutes than other students who study in inner areas do. This issue should be further investigated.

We do some robustness checks, the results of which are reported in S3 Appendix. By excluding students who live outside Bangkok, we obtain results consistent with the main findings. In addition, we separately run regressions of commuting distance and commuting time; the results are robust. These results, however, should be interpreted with caution, because they are based on a small number of observations.

The results of students in other provinces (Panel B of Table 2) are slightly different from those in Bangkok. Both commuting distance and time affect students’ mental health. On average, an increase in one kilometer of commuting distance and one hour of commuting time increases the chance of having a low level of mental impairment by 0.01% and 4.05%, respectively. The coefficient magnitudes of commuting distance are far smaller than commuting time. This indicates the strong impacts on students’ health of the duration of their commute to school.

Considering commuting effects on physical health in Panel A of Table 3, students in Bangkok are likely to experience physical health issues as commuting time increases. Spending an additional hour on the road increases the probability of having low to medium physical health issues by 12.17% and 9.77%, respectively. Commuting distance is insignificant, indicating no effect on students’ physical health.

**Table 3:** Commuting effects on physical health.

	Coefficient	Marginal Effects			
		*Y*_*i*_ = 0	*Y*_*i*_ = 1	*Y*_*i*_ = 2	*Y*_*i*_ = 3
		No effect	Low	Medium	High
**A: Bangkok**					
Distance	-0.0230	0.0048	-0.0026	-0.0021	-0.0002
	(0.0150)	(0.0031)	(0.0017)	(0.0013)	(0.0001)
Time	1.0933***	-0.2282***	0.1217***	0.0977***	0.0088***
	(0.1622)	(0.0321)	(0.0189)	(0.0154)	(0.0030)
Thresholds					
*Y*_*i*_ = 1 (Threshold1)	2.5614	Observations	961		
*Y*_*i*_ = 2 (Threshold2)	4.3288	R^2^	0.0835		
*Y*_*i*_ = 3 (Threshold3)	7.3277				
**B: Other provinces**					
Distance	0.0237***	-0.0016***	0.0013***	0.0003***	0.0000***
	(0.0041)	(0.0003)	(0.0002)	(0.0000)	(0.0000)
Time	0.6752***	-0.0451***	0.0362***	0.0075***	0.0013***
	(0.0867)	(0.0058)	(0.0047)	(0.0011)	(0.0003)
Thresholds					
*Y*_*i*_ = 1 (Threshold1)	1.9106	Observations	24,500		
*Y*_*i*_ = 2 (Threshold2)	3.8870	R^2^	0.1309		
*Y*_*i*_ = 3 (Threshold3)	5.8627				

**Notes:** In the ordered logistic regression, the well-being rating ranges from zero to three, where zero indicates no negative effect and three indicates a high negative effect on health. Robust standard errors are in parentheses. The full results of the ordered logistic regression are reported in S2 Appendix.

*p < 0.10

**p < 0.05

***p < 0.01.

Estimated physical health equal to zero indicates no effect. The thresholds show that estimated physical health ranges between more than zero and less than or equal to Threshold1 ($0<\hat{Y_{i}}\leq Threshold1$) indicate a low effect on physical health (*Y*_*i*_ = 1). Estimated physical health ranges between more than Threshold1 and less than or equal to Threshold2 ($Threshold1<\hat{Y_{i}}\leq Threshold2$) indicate a medium effect on physical health (*Y*_*i*_ = 2). Estimated physical health ranges between more than Threshold2 and less than or equal to Threshold3 ($Threshold2<\hat{Y_{i}}\leq Threshold3$) indicate a high effect on physical health (*Y*_*i*_ = 3).

In other provinces, both commuting distance and time highly impact students’ physical health. An increased commuting distance stimulates low to medium physical health issues by 0.13% and 0.03%, respectively. Similarly, a one-hour increase in commuting time to school increases in low- and medium-level physical issues by 3.62% and 0.75%, respectively.

To take household locations into account, we subdivide the data into different school-household locations. Since the number of students in Bangkok is small, leading to inaccurate results, we exclude all students in Bangkok from this analysis.

The results in Table 4 indicate that, overall, increases in commuting durations and distances significantly stimulate mental health issues (Panels A, B, and D). For students studying in urban areas, both commuting distance and time significantly cause high levels of mental health issues. Only students who commute outbound—living in municipalities and studying in nonmunicipalities—are unaffected by increased commuting distance and time (Panel C).

**Table 4:** Commuting effects on mental health by school-home location in other provinces.

	Coefficient	Marginal Effects			
		No effect	Low	Medium	High
**A: Municipality-Municipality**					
Distance	0.0115*	-0.0009*	0.0007*	0.0002*	0.0000*
	(0.0061)	(0.0005)	(0.0004)	(0.0001)	(0.0000)
Time	0.8563***	-0.0700***	0.0537***	0.0146***	0.0017***
	(0.1286)	(0.0105)	(0.0080)	(0.0024)	(0.0004)
Thresholds		Observations	11,525		
*Y*_*i*_ = 1 (Threshold1)	1.8904	R^2^	0.1612		
*Y*_*i*_ = 2 (Threshold2)	3.8303				
*Y*_*i*_ = 3 (Threshold3)	6.2112				
**B: Municipality-Nonmunicipality**					
Distance	0.0159**	-0.0017**	0.0013**	0.0004**	0.0000**
	(0.0066)	(0.0007)	(0.0005)	(0.0002)	(0.0000)
Time	0.5811***	-0.0620***	0.0467***	0.0138***	0.0014**
	(0.1325)	(0.0141)	(0.0106)	(0.0033)	(0.0006)
Thresholds		Observations	4,808		
*Y*_*i*_ = 1 (Threshold1)	2.0110	R^2^	0.1528		
*Y*_*i*_ = 2 (Threshold2)	4.0082				
*Y*_*i*_ = 3 (Threshold3)	6.5356				
**C: Nonmunicipality- Municipality**					
Distance	-0.0029	0.0002	-0.0002	0.0000	0.0000
	(0.0339)	(0.0026)	(0.0020)	(0.0005)	(0.0000)
Time	0.9480	-0.0720	0.0567	0.0142	0.0011
	(0.5878)	(0.0451)	(0.0364)	(0.0090)	(0.0012)
Thresholds		Observations	750		
*Y*_*i*_ = 1 (Threshold1)	2.1311	R^2^	0.2262		
*Y*_*i*_ = 2 (Threshold2)	4.2856				
*Y*_*i*_ = 3 (Threshold3)	7.4520				
**D: Nonmunicipality-Nonmunicipality**					
Distance	0.0284**	-0.0013**	0.0012**	0.0001**	0.0000
	(0.0115)	(0.0005)	(0.0005)	(0.0001)	(0.0000)
Time	0.4427**	-0.0206**	0.0184**	0.0021**	0.0001
	(0.2101)	(0.0098)	(0.0088)	(0.0011)	(0.0001)
Thresholds		Observations	7,417		
*Y*_*i*_ = 1 (Threshold1)	2.3716	R^2^	0.1749		
*Y*_*i*_ = 2 (Threshold2)	4.9053				
*Y*_*i*_ = 3 (Threshold3)	7.8734				

Notes: The observations reported in Table 4 exclude students living in Bangkok. In the ordered logistic regression, the well-being rating ranges from zero to three, in which zero indicates no negative effect and three indicates a high negative effect on health. Robust standard errors are in parentheses.

*p < 0.10

**p < 0.05

***p < 0.01.

Estimated physical health equal to zero indicates no effect. The thresholds show that estimated physical health ranges between more than zero and less than or equal to Threshold1 ($0<\hat{Y_{i}}\leq Threshold1$) indicate a low effect on physical health (*Y*_*i*_ = 1). Estimated physical health ranges between more than Threshold1 and less than or equal to hTreshold2 ($Threshold1<\hat{Y_{i}}\leq Threshold2$) indicate a medium effect on physical health (*Y*_*i*_ = 2). Estimated physical health ranges between more than Threshold2 and less than or equal to Threshold3 ($Threshold2<\hat{Y_{i}}\leq Threshold3$) indicate a high effect on physical health (*Y*_*i*_ = 3).

The coefficients of commuting distance are smaller than commuting time for almost all school-household locations.

The results in Table 5 regarding the impact of school commuting on physical health are similar to those on mental health. Commuting duration to school is still an important factor, leading to physical health issues for young students. Students studying in municipalities can experience higher negative physical issues than those studying in nonmunicipalities.

**Table 5:** Commuting effects on physical health by school-home location in other provinces.

	Coefficient	Marginal Effects			
		No effect	Low	Medium	High
**A: Municipality-Municipality**					
Distance	0.0213***	-0.0014***	0.0011***	0.0003***	0.0001***
	(0.0064)	(0.0004)	(0.0003)	(0.0001)	(0.0000)
Time	0.8839***	-0.0586***	0.0459***	0.0106***	0.0022***
	(0.1393)	(0.0093)	(0.0073)	(0.0019)	(0.0005)
Thresholds		Observations	11,525		
*Y*_*i*_ = 1 (Threshold1)	1.9137	R^2^	0.1473		
*Y*_*i*_ = 2 (Threshold2)	3.8115				
*Y*_*i*_ = 3 (Threshold3)	5.6727				
**B: Municipality-Nonmunicipality**					
Distance	0.0216***	-0.0019***	0.0015***	0.0004***	0.0001**
	(0.0069)	(0.0006)	(0.0005)	(0.0001)	(0.0000)
Time	0.5228***	-0.0468***	0.0360***	0.0094***	0.0014***
	(0.1450)	(0.0130)	(0.0100)	(0.0027)	(0.0006)
Thresholds		Observations	4,808		
*Y*_*i*_ = 1 (Threshold1)	2.0183	R^2^	0.1617		
*Y*_*i*_ = 2 (Threshold2)	4.0239				
*Y*_*i*_ = 3 (Threshold3)	6.2121				
**C: Nonmunicipality-Municipality**					
Distance	-0.0520*	0.0035*	-0.0029*	-0.0004	-0.0002
	(0.0308)	(0.0021)	(0.0017)	(0.0003)	(0.0002)
Time	1.7610***	-0.1183***	0.0975***	0.0140**	0.0068
	(0.6469)	(0.0438)	(0.0367)	(0.0071)	(0.0047)
Thresholds		Observations	750		
*Y*_*i*_ = 1 (Threshold1)	2.1323	R^2^	0.2429		
*Y*_*i*_ = 2 (Threshold2)	4.5202				
*Y*_*i*_ = 3 (Threshold3)	5.8104				
**D: Nonmunicipality-Nonmunicipality**					
Distance	0.0212*	-0.0010*	0.0010*	0.0000*	0.0000
	(0.0116)	(0.001)	(0.001)	(0.000)	(0.000)
Time	0.5085**	-0.0230**	0.0200**	0.0030**	0.0000
	(0.2052)	(0.009)	(0.008)	(0.001)	(0.000)
Thresholds		Observations	7,417		
*Y*_*i*_ = 1 (Threshold1)	1.9283	R^2^	0.1488		
*Y*_*i*_ = 2 (Threshold2)	4.2864				
*Y*_*i*_ = 3 (Threshold3)	6.6618				

Notes: The observations reported in Table 5 exclude students living in Bangkok. In the ordered logistic regression, the well-being rating ranges from zero to three, in which zero indicates no negative effect and three indicates a high negative effect on health. Robust standard errors are in parentheses.

*p < 0.10

**p < 0.05

***p < 0.01.

Estimated physical health that is equal to zero indicates no effect. The thresholds show that estimated physical health ranges between more than zero and less than or equal to Threshold1 ($0<\hat{Y_{i}}\leq Threshold1$) indicate a low effect on physical health (*Y*_*i*_ = 1). Estimated physical health ranges between more than Threshold1 and less than or equal to Threshold2 ($Threshold1<\hat{Y_{i}}\leq Threshold2$) indicate a medium effect on physical health (*Y*_*i*_ = 2). Estimated physical health ranges between more than Threshold2 and less than or equal to Threshold3 ($Threshold2<\hat{Y_{i}}\leq Threshold3$) indicate a high effect on physical health (*Y*_*i*_ = 3).

Commuting distance significantly affects students who study in urban areas. An increase of one kilometer of distance to school can stimulate low negative physical issues by approximately 0.10% for those students.

## 5. Study limitations

There are data limitations in this study. First, according to the NSO’s questions, our study can assess only negative commuting-to-school impacts on physical and mental health issues. Second, by using self-assessment questions, which are subjectively measured, the trip-specific influences on health may differ from day to day [26] and cannot be considered steady states. Additionally, commuting time and distance are self-estimated, which means they may be overestimated or underestimated. However, they are considered to be appropriate proxies when actual data are unavailable [10].

## 6. Conclusions and discussion

Distance and duration of commute to and from school can impact student’s well-being, increasing concern by parents and officials. We jointly investigate the effects of both commuting distance and time on students’ health. According to our results, three key points should be discussed as follows:

1. Students in Thailand physically and mentally suffer from long commuting distances and times. Our results are consistent with those of other previous studies (e.g., [9, 11]). These results reaffirm that the impacts of long commutes on children’s health in Thailand, a developing country, are not different from those in developed countries and those of adults (e.g., [3, 27]).

Traffic in Bangkok has recently become worse. Commuting in heavy traffic can take at least 30 minutes for a one-way trip of less than 5 kilometers [28]. The commuting speed on average is approximately 25 kilometers per hour [22]. Our results from this study may indicate the lower bound of current health impacts from school commutes for children.

2. Only students who commute outbound to study in rural areas are physically, not mentally, affected by the school commute. [2] find that students in rural areas commute longer distances to school than urban students, causing rural students to have mental health impairment.

Our data also show that outbound commutes to study in rural areas take the longest distance compared to other commutes. However, those students who commute outbound have the second highest distance per hour, implying light traffic, which insignificantly affects students’ mental health. Positive mental health—i.e., emotion or commuting satisfaction—can be a result of involvement in activities during the commute to school, such as social activities and surrounding enjoyment (see [29]). Long school commutes may encourage students to get involved in those activities. Long-distance commutes, however, may cause physical exhaustion, such as being seated for a long time.

On the other hand, traffic limits social interactions [29] and is possibly a main cause of mental impairment for students who study or live, or both, in Bangkok and municipal areas.

3. Both commuting distance and time jointly affect students’ health.

Our results emphasize different traffic issues and commuting structures across areas, such as public transportation and road quality, which may explain the low correlation between commuting distance and commuting time. Previous studies concern the high correlations between commuting distance and time, leading them to investigate the effects of commuting based on either of those variables. Omitting one of them, however, may bias the results. This study can be applied to areas with traffic and infrastructure conditions, such as most cities in Southeast Asian countries with heavy traffic, inadequate public transportation, and no specific policy to control the number of private cars.

## 7. Recommendations

### 7.1 Policy implications

Commuting duration is an essential factor in physical and mental health issues. One suggestion, which may be difficult to implement, is improving traffic to reduce commuting time, particularly in Bangkok and urban areas. Another suggestion is incentivizing parents to choose a school close to their home. The Thai government has encouraged public schools to increase enrollment quotas of students living close to schools. Without city planning, only a few households, particularly in Bangkok, are located close to schools within walking distance—according to our data, only 12% of students in Bangkok walk to school. Additionally, Thai parents have a preference for well-known and reputable schools. All these factors deter students from having a shorter school commute.

Specifically in rural areas, our results point out that commuting time and distance affect the physical and mental health issues of students. Since 2020, the Thai government has started implementing the small-school-merging policy to reduce the subsidies to small public schools [30]. This policy can require students to commute longer distances to school, for a longer duration, especially in rural and remote areas.

### 7.2 Recommendations for further studies

Future studies should investigate this issue. In rural areas, even though students commute shorter distances than those in urban areas do, the commuting distance to school determines students’ health issues. This may be related to basic structures, such as road quality, for which we do not have enough data. Future studies should explore such issues that may affect the physical and mental health of students in rural areas. In addition, the effects of school consolidations, particularly in rural areas, should be further observed since the measures may increase commuting duration and distance.

## Supporting information

S1 Appendix Correlation between commuting distance and time. (DOCX)

S2 Appendix Full regression results. (DOCX)

S3 Appendix Additional analyses (excluding students living outside Bangkok). (DOCX)
