# Supplementary material for: Getting there: How commuting time and distance impact students’ health
Source: PLoS One. 2024 Dec 6;19(12):e0314687. doi: 10.1371/journal.pone.0314687 (PMC11623559; doi:10.1371/journal.pone.0314687)
Supplement: S1 Appendix — (DOCX) [file pone.0314687.s001.docx]

**Appendix A** Correlation between commuting distance and time

|  |  | **Commuting time** |
| --- | --- | --- |
| **Commuting distance** | **Bangkok** | 0.5808 |
|  | **Other province** | 0.7038 |
