# Supplementary material for: Getting there: How commuting time and distance impact students’ health
Source: PLoS One. 2024 Dec 6;19(12):e0314687. doi: 10.1371/journal.pone.0314687 (PMC11623559; doi:10.1371/journal.pone.0314687)
Supplement: S2 Appendix — (DOCX) [file pone.0314687.s002.docx]

**Appendix B** Full regression results

**Table B1** Commuting Effects on Mental Health

|  | **Coefficient** | **Marginal Effects** | | | |
| --- | --- | --- | --- | --- | --- |
|  |  | $\boldsymbol{Y}_{\boldsymbol{i}}$**=0** | $\boldsymbol{Y}_{\boldsymbol{i}}$**=1** | $\boldsymbol{Y}_{\boldsymbol{i}}$**=2** | $\boldsymbol{Y}_{\boldsymbol{i}}$**=3** |
|  |  | **No effect** | **Low** | **Medium** | **High** |
| **A: Bangkok** |  |  |  |  |  |
| Distance | -0.0262* | 0.0055* | -0.0026* | -0.0027* | -0.0002 |
|  | (0.0152) | (0.0032) | (0.0016) | (0.0016) | (0.0001) |
| Time | 1.1206*** | -0.2349*** | 0.1121*** | 0.1150*** | 0.0077*** |
|  | (0.1649) | (0.0328) | (0.0175) | (0.0178) | (0.0029) |
| Female | 0.0398 | -0.0084 | 0.0040 | 0.0041 | 0.0003 |
|  | (0.1359) | (0.0285) | (0.0136) | (0.0140) | (0.0010) |
| Age | 0.0959*** | -0.0201*** | 0.0096*** | 0.0098*** | 0.0007** |
|  | (0.0266) | (0.0055) | (0.0026) | (0.0028) | (0.0003) |
| Number of household members | -0.0098 | 0.0021 | -0.0010 | -0.0010 | -0.0001 |
|  | (0.0411) | (0.0086) | (0.0041) | (0.0042) | (0.0003) |
| Family income | 0.0030 | -0.0006 | 0.0003 | 0.0003 | 0.0000 |
|  | (0.0019) | (0.0004) | (0.0002) | (0.0002) | (0.0000) |
| Commuting at rush hour | -0.1834 | 0.0384 | -0.0183 | -0.0188 | -0.0013 |
|  | (0.1546) | (0.0323) | (0.0154) | (0.0159) | (0.0012) |
| Active commuting mode | -0.9839*** | 0.2062*** | -0.0984*** | -0.1010*** | -0.0068** |
|  | (0.3429) | (0.0707) | (0.0337) | (0.0357) | (0.0035) |
| Thresholds |  |  |  |  |  |
| $Y_{i}$=1 (Threshold1) | 2.2602 | Observations | 961 |  |  |
| $Y_{i}$=2 (Threshold2) | 3.9964 | R^2^ | 0.0975 |  |  |
| $Y_{i}$=3 (Threshold3) | 7.4277 |  |  |  |  |
| **B: Other provinces** | | | | | |
| Distance | 0.0193*** | -0.0015*** | 0.0012*** | 0.0003*** | 0.0000*** |
|  | (0.0039) | (0.0003) | (0.0002) | (0.0001) | (0.0000) |
| Time | 0.6531*** | -0.0513*** | 0.0405*** | 0.0098*** | 0.0010*** |
|  | (0.0797) | (0.0063) | (0.005) | (0.0013) | (0.0002) |
| Female | 0.0113 | -0.0009 | 0.0007 | 0.0002 | 0.0000 |
|  | (0.0463) | (0.0036) | (0.0029) | (0.0007) | (0.0001) |
| Age | 0.0426*** | -0.0034*** | 0.0026*** | 0.0006*** | 0.0001*** |
|  | (0.0094) | (0.0007) | (0.0006) | (0.0001) | (0.0000) |
| Number of household members | -0.0056 | 0.0004 | -0.0003 | -0.0001 | -0.0000 |
|  | (0.0130) | (0.0010) | (0.0008) | (0.0002) | (0.0000) |
| Family income | 0.0054*** | -0.0004*** | 0.0003*** | 0.0001*** | 0.0000*** |
|  | (0.0015) | (0.0001) | (0.0001) | (0.0000) | (0.0000) |
| Commuting at rush hour | -0.1992*** | 0.0157 | -0.0124*** | -0.0030*** | -0.0003** |
|  | (0.0756) | (0.0059) | (0.0047) | (0.0011) | (0.0001) |
| Active commuting mode | -0.7989*** | 0.0628*** | -0.0496*** | -0.0119*** | -0.0012*** |
|  | (0.1020) | (0.0080) | (0.0063) | (0.0016) | (0.0003) |
| Thresholds |  |  |  |  |  |
| $Y_{i}$=1 (Threshold1) | 1.9991 | Observations | 24,500 |  |  |
| $Y_{i}$=2 (Threshold2) | 3.9882 | R^2^ | 0.1412 |  |  |
| $Y_{i}$=3 (Threshold3) | 6.4551 |  |  |  |  |

**Notes:** In the ordered logistic regression, the well-being rating ranges from zero to three, in which zero indicates no negative effect and three indicates a high negative effect on health. Robust standard errors are in parentheses. *p < 0.10; **p < 0.05; ***p < 0.01.

Estimated mental health equal to zero indicates no effect. The thresholds show that estimated mental health ranges between more than zero and less than or equal to Threshold1 ($0<\hat{Y_{i}}\leq Threshold1$) indicate a low effect on mental health ($Y_{i}=1$). Estimated mental health ranges between more than Threshold1 and less than or equal to Threshold2 ($Threshold1<\hat{Y_{i}}\leq Threshold2$) indicate a medium effect on mental health ($Y_{i}=2$). Estimated mental health ranges between more than Threshold2 and less than or equal to Threshold3 ($Threshold2<\hat{Y_{i}}\leq Threshold3$) indicate a high effect on mental health ($Y_{i}=3$).

**Table B2** Commuting Effects on Physical Health

|  | **Coefficient** | **Marginal Effects** | | | |
| --- | --- | --- | --- | --- | --- |
|  |  | $\boldsymbol{Y}_{\boldsymbol{i}}$**=0** | $\boldsymbol{Y}_{\boldsymbol{i}}$**=1** | $\boldsymbol{Y}_{\boldsymbol{i}}$**=2** | $\boldsymbol{Y}_{\boldsymbol{i}}$**=3** |
|  |  | **No effect** | **Low** | **Medium** | **High** |
| **A: Bangkok** |  |  |  |  |  |
| Distance | -0.0230 | 0.0048 | -0.0026 | -0.0021 | -0.0002 |
|  | (0.0150) | (0.0031) | (0.0017) | (0.0013) | (0.0001) |
| Time | 1.0933*** | -0.2282*** | 0.1217*** | 0.0977*** | 0.0088*** |
|  | (0.1622) | (0.0321) | (0.0189) | (0.0154) | (0.0030) |
| Female | 0.0252 | -0.0053 | 0.0028 | 0.0023 | 0.0002 |
|  | (0.1369) | (0.0286) | (0.0152) | (0.0122) | (0.0011) |
| Age | 0.0829*** | -0.0173*** | 0.0092*** | 0.0074*** | 0.0007** |
|  | (0.0271) | (0.0056) | (0.0030) | (0.0025) | (0.0003) |
| Number of household members | 0.0023 | -0.0005 | 0.0003 | 0.0002 | 0.0000 |
|  | (0.0396) | (0.0083) | (0.0044) | (0.0035) | (0.0003) |
| Family income | 0.0055*** | -0.0011*** | 0.0006*** | 0.0005*** | 0.0000** |
|  | (0.0019) | (0.0004) | (0.0002) | (0.0002) | (0.0000) |
| Commuting at rush hour | -0.0944 | 0.0197 | -0.0105 | -0.0084 | -0.0008 |
|  | (0.1576) | (0.0329) | (0.0175) | (0.0142) | (0.0013) |
| Active commuting mode | -0.6858** | 0.1432** | -0.0764** | -0.0613** | -0.0055 |
|  | (0.3427) | (0.0710) | (0.0379) | (0.0308) | (0.0034) |
| Thresholds |  |  |  |  |  |
| $Y_{i}$=1 (Threshold1) | 2.5614 | Observations | 961 |  |  |
| $Y_{i}$=2 (Threshold2) | 4.3288 | R^2^ | 0.0835 |  |  |
| $Y_{i}$=3 (Threshold3) | 7.3277 |  |  |  |  |
| **B: Other Provinces** | | | | | |
| Distance | 0.0237*** | -0.0016*** | 0.0013*** | 0.0003*** | 0.0000*** |
|  | (0.0041) | (0.0003) | (0.0002) | (0.0000) | (0.0000) |
| Time | 0.6752*** | -0.0451*** | 0.0362*** | 0.0075*** | 0.0013*** |
|  | (0.0867) | (0.0058) | (0.0047) | (0.0011) | (0.0003) |
| Female | 0.0806 | -0.0054 | 0.0043 | 0.0009 | 0.0002 |
|  | (0.0507) | (0.0034) | (0.0027) | (0.0006) | (0.0001) |
| Age | 0.0304*** | -0.0020*** | 0.0016*** | 0.0003*** | 0.0001*** |
|  | (0.0103) | (0.0007) | (0.0006) | (0.0001) | (0.0000) |
| Number of household members | -0.0109 | 0.0007 | -0.0006 | -0.0001 | -0.0000 |
|  | (0.0152) | (0.0010) | (0.0008) | (0.0002) | (0.0000) |
| Family income | 0.0019 | -0.0001 | 0.0001 | 0.0000 | 0.0000 |
|  | (0.0018) | (0.0001) | (0.0001) | (0.0000) | (0.0000) |
| Commuting at rush hour | -0.1370* | 0.0091 | -0.0073* | -0.0015 | -0.0003 |
|  | (0.0831) | (0.0056) | (0.0045) | (0.0009) | (0.0002) |
| Active commuting mode | -0.3915***  (0.1054) | 0.0261***  (0.0070) | -0.0210***  (0.0057) | -0.0044***  (0.0012) | -0.0008***  (0.0002) |
| Thresholds |  |  |  |  |  |
| $Y_{i}$=1 (Threshold1) | 1.9106 | Observations | 24,500 |  |  |
| $Y_{i}$=2 (Threshold2) | 3.8870 | R^2^ | 0.1309 |  |  |
| $Y_{i}$=3 (Threshold3) | 5.8627 |  |  |  |  |

**Notes:** In the ordered logistic regression, the well-being rating ranges from zero to three, where zero indicates no negative effect and three indicates a high negative effect on health. Robust standard errors are in parentheses. *p < 0.10; **p < 0.05; ***p < 0.01.

Estimated mental/physical health equal to zero indicates no effect. The thresholds show that estimated mental/physical health ranges between more than zero and less than or equal to Threshold1 ($0<\hat{Y_{i}}\leq Threshold1$) indicate a low effect on mental/physical health ($Y_{i}=1$). Estimated mental/physical health ranges between more than Threshold1 and less than or equal to Threshold2 ($Threshold1<\hat{Y_{i}}\leq Threshold2$) indicate a medium effect on physical health ($Y_{i}=2$). Estimated mental/physical health ranges between more than Threshold2 and less than or equal to Threshold3 ($Threshold2<\hat{Y_{i}}\leq Threshold3$) indicate a high effect on mental/physical health ($Y_{i}=3$).
