# Supplementary material for: Getting there: How commuting time and distance impact students’ health
Source: PLoS One. 2024 Dec 6;19(12):e0314687. doi: 10.1371/journal.pone.0314687 (PMC11623559; doi:10.1371/journal.pone.0314687)
Supplement: S3 Appendix — (DOCX) [file pone.0314687.s003.docx]

**Appendix C** Additional analyses (excluding students living outside Bangkok)

|  | **Coefficient** | **Marginal Effects** | | | |
| --- | --- | --- | --- | --- | --- |
|  |  | $\boldsymbol{Y}_{\boldsymbol{i}}$**=0** | $\boldsymbol{Y}_{\boldsymbol{i}}$**=1** | $\boldsymbol{Y}_{\boldsymbol{i}}$**=2** | $\boldsymbol{Y}_{\boldsymbol{i}}$**=3** |
|  |  | **No effect** | **Low** | **Medium** | **High** |
| **A: Mental health** |  |  |  |  |  |
| Distance | -0.0306* | 0.0065* | -0.0033* | -0.0030* | -0.0002 |
|  | (0.0161) | (0.0034) | (0.0018) | (0.0016) | (0.0001) |
| Time | 1.1665*** | -0.2474*** | 0.1250*** | 0.1160*** | 0.0064** |
|  | (0.1668) | (0.0331) | (0.0188) | (0.0171) | (0.0029) |
| Thresholds |  |  |  |  |  |
| $Y_{i}$=1 (Threshold1) | 2.3075 | Observations | 911 |  |  |
| $Y_{i}$=2 (Threshold2) | 4.0616 | R^2^ | 0.0792 |  |  |
| $Y_{i}$=3 (Threshold3) | 7.4572 |  |  |  |  |
| **B: Physical health** |  |  |  |  |  |
| Distance | -0.0344** | 0.0072** | -0.0042** | -0.0029** | -0.0002 |
|  | (0.0163) | (0.0034) | (0.0020) | (0.0013) | (0.0001) |
| Time | 1.1716*** | -0.2466*** | 0.1418*** | 0.0972*** | 0.0076** |
|  | (0.1651) | (0.0323) | (0.0206) | (0.0145) | (0.0032) |
| Thresholds |  |  |  |  |  |
| $Y_{i}$=1 (Threshold1) | 2.5340 | Observations | 911 |  |  |
| $Y_{i}$=2 (Threshold2) | 4.3671 | R^2^ | 0.0029 |  |  |
| $Y_{i}$=3 (Threshold3) | 7.3361 |  |  |  |  |

**Notes:** In the ordered logistic regression, the well-being rating ranges from zero to three, where zero indicates no negative effect and three indicates a high negative effect on health. Robust standard errors are in parentheses. *p < 0.10; **p < 0.05; ***p < 0.01.

Estimated physical health equal to zero indicates no effect. The thresholds show that estimated physical health ranges between more than zero and less than or equal to Threshold1 ($0<\hat{Y_{i}}\leq Threshold1$) indicate a low effect on physical health ($Y_{i}=1$). Estimated physical health ranges between more than Threshold1 and less than or equal to Threshold2 ($Threshold1<\hat{Y_{i}}\leq Threshold2$) indicate a medium effect on physical health ($Y_{i}=2$). Estimated physical health ranges between more than Threshold2 and less than or equal to Threshold3 ($Threshold2<\hat{Y_{i}}\leq Threshold3$) indicate a high effect on physical health ($Y_{i}=3$).
